# Development and Validation of a Novel Methodological Pipeline to Integrate Neuroimaging and Photogrammetry for Immersive 3D Cadaveric Neurosurgical Simulation

**DOI:** 10.3389/fsurg.2022.878378

**Published:** 2022-05-16

**Authors:** Sahin Hanalioglu, Nicolas Gonzalez Romo, Giancarlo Mignucci-Jiménez, Osman Tunc, Muhammet Enes Gurses, Irakliy Abramov, Yuan Xu, Balkan Sahin, Ilkay Isikay, Ilkan Tatar, Mustafa Berker, Michael T. Lawton, Mark C. Preul

**Affiliations:** ^1^ Department of Neurosurgery, Barrow Neurological Institute, St. Joseph’s Hospital and Medical Center, Phoenix, Arizona; ^2^ Department of Neurosurgery, Hacettepe University Faculty of Medicine, Ankara, Turkey; ^3^ BTech Innovation, METU Technopark, Ankara, Turkey; ^4^ Department of Neurosurgery, University of Health Sciences, Sisli Hamidiye Etfal Training and Research Hospital, Istanbul, Turkey; ^5^ Department of Anatomy, Hacettepe University Faculty of Medicine, Ankara, Turkey

**Keywords:** 3D rendering, depth estimation, neuroanatomy, neuroimaging, neurosurgical training, photogrammetry, virtual model

## Abstract

**Background:**

Visualizing and comprehending 3-dimensional (3D) neuroanatomy is challenging. Cadaver dissection is limited by low availability, high cost, and the need for specialized facilities. New technologies, including 3D rendering of neuroimaging, 3D pictures, and 3D videos, are filling this gap and facilitating learning, but they also have limitations. This proof-of-concept study explored the feasibility of combining the spatial accuracy of 3D reconstructed neuroimaging data with realistic texture and fine anatomical details from 3D photogrammetry to create high-fidelity cadaveric neurosurgical simulations.

**Methods:**

Four fixed and injected cadaver heads underwent neuroimaging. To create 3D virtual models, surfaces were rendered using magnetic resonance imaging (MRI) and computed tomography (CT) scans, and segmented anatomical structures were created. A stepwise pterional craniotomy procedure was performed with synchronous neuronavigation and photogrammetry data collection. All points acquired in 3D navigational space were imported and registered in a 3D virtual model space. A novel machine learning-assisted monocular-depth estimation tool was used to create 3D reconstructions of 2-dimensional (2D) photographs. Depth maps were converted into 3D mesh geometry, which was merged with the 3D virtual model’s brain surface anatomy to test its accuracy. Quantitative measurements were used to validate the spatial accuracy of 3D reconstructions of different techniques.

**Results:**

Successful multilayered 3D virtual models were created using volumetric neuroimaging data. The monocular-depth estimation technique created qualitatively accurate 3D representations of photographs. When 2 models were merged, 63% of surface maps were perfectly matched (mean [SD] deviation 0.7 ± 1.9 mm; range −7 to 7 mm). Maximal distortions were observed at the epicenter and toward the edges of the imaged surfaces. Virtual 3D models provided accurate virtual measurements (margin of error <1.5 mm) as validated by cross-measurements performed in a real-world setting.

**Conclusion:**

The novel technique of co-registering neuroimaging and photogrammetry-based 3D models can (1) substantially supplement anatomical knowledge by adding detail and texture to 3D virtual models, (2) meaningfully improve the spatial accuracy of 3D photogrammetry, (3) allow for accurate quantitative measurements without the need for actual dissection, (4) digitalize the complete surface anatomy of a cadaver, and (5) be used in realistic surgical simulations to improve neurosurgical education.

## Introduction

One of the most challenging aspects of neurosurgery is visualizing and comprehending 3-dimensional (3D) neuroanatomy. The acquisition of this knowledge and its successful clinical application require years of practice in the operating room and anatomical dissection laboratory. However, both of these training resources may be limited by institutional resources. Furthermore, residents’ work-hour restrictions, decreases in cadaver availability, and the interruption of hands-on training courses as a result of the COVID-19 pandemic have increased the difficulty of acquiring appropriate anatomical training for neurosurgeons. However, the advent of new technology that enables 3D rendering of neuroimaging has allowed the development of 3D virtual models that can be used to augment neurosurgical education. Such tools have been shown to improve performance and have become a complementary part of neurosurgical education when access to cadaveric specimens is restricted and will likely achieve more integration and impact upon neurosurgical training (1–4).

New advances in imaging, computer vision, image-processing technologies, and multidimensional rendering of neuroanatomical models have introduced extended-reality educational tools, such as virtual and augmented reality, into neurosurgeon training (2–6). Photogrammetry and related technologies may complement advanced tomographic neuroimaging studies (e.g., magnetic resonance imaging [MRI] and computed tomography [CT]) by providing detailed surface information of neuroanatomical structures. Stereoscopy, which is commonly used for neurosurgical education (7–9), is the process by which two 2-dimensional (2D) photographs of the same object taken at slightly different angles are viewed together, creating an impression of depth and solidity. Photogrammetry is a technique in which 2D photographs of an object are taken at varying angles (up to 360°) and then overlaid using computer software to generate a 3D reconstruction (10). In a previous study (1), a 3D model of a real cadaveric brain specimen was created using photogrammetry and 360° spanning. Although this technique is very practical and useful, it is limited to objects that can be scanned in 360°. Unfortunately, in most surgical scenarios, the surgeon or trainee can only visualize a portion of the surgical anatomy, usually through a narrow corridor. Thus, more practical photogrammetric approaches are needed to overcome this barrier. Recently, an artificial intelligence (AI)-based tool was developed to estimate monocular depth, which potentially makes it possible to convert 2D photographs into 3D images. We believe that this tool can be used to overcome limitations and bridge the gap between neuroimaging and photogrammetry scanning technologies.

In this proof-of-concept study, we brought together the spatial accuracy of 3D reconstruction of cadaveric neuroimaging data with the realistic texture of 3D photogrammetry to create high-fidelity neurosurgical simulations for education and surgical planning. The method sought to merge the spatial accuracy of medical MRI and CT images with 3D textural features obtained using advanced photogrammetry techniques. We hypothesized that current imaging, photography, computer vision, and rendering technologies could be effectively combined to maximize the use of cadaveric specimens for education, surgical planning, and research. This innovative approach aimed to not only enhance neurosurgical anatomy training but also create a new digital interactive cadaveric imaging database for future anatomical research. If this attempt is successful, a cadaveric specimen would no longer have to be discarded after fixation and dissection. A cadaver could instead be digitalized and passed along from generation to generation in the same condition in which it was first dissected. Herein, we describe and validate our novel methodological paradigm to create qualitatively and quantitatively accurate 3D models.

## Materials and Methods

### Study Design

**Figure 1:**
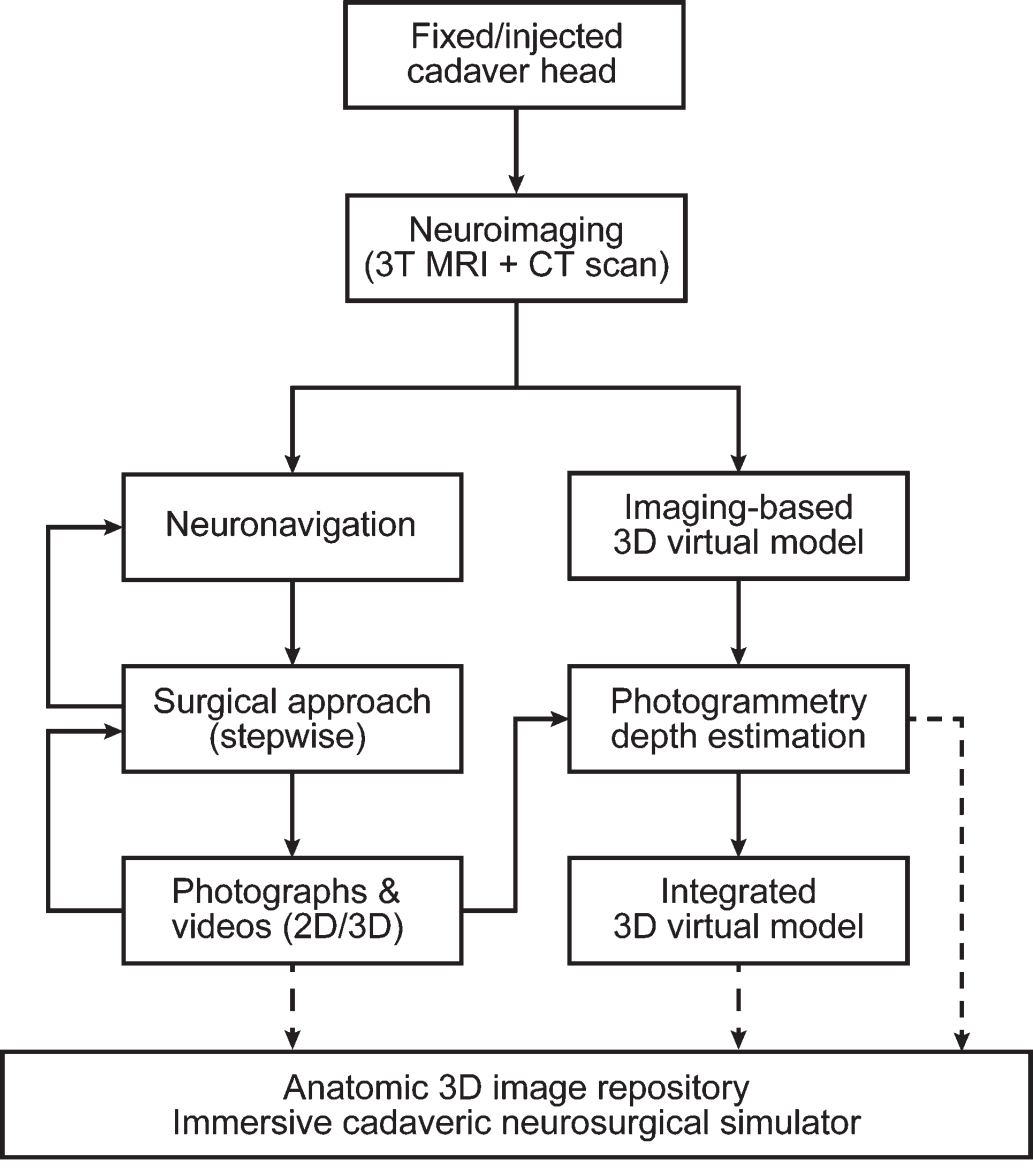
The methodological pipeline to fully digitalize the cadavers. *Used with permission from Barrow Neurological Institute, Phoenix, Arizona*.

No institutional review board approval was required for this study. This proof-of-concept study included two phases: development and validation. In the first phase, a methodological pipeline was developed to combine various imaging, modeling, and visualization techniques to create integrated, multilayered, immersive 3D models for cadaveric research and education (Figure 1). After the development phase, the second phase of the study included validation of this novel methodological approach by matching agreement between surface maps. Four embalmed and injected cadaver heads were used for method development (*n* = 3) and quantitative validation (*n* = 1). We initially used 3 heads to optimize the methodological pipeline. Once optimized, we applied this pipeline to produce the virtual model in 1 head for this proof-of-concept study. Importantly, all heads used in this study were of the highest anatomical quality.

### Neuroimaging

All cadaver heads underwent MRI and CT scans. A 3T MR-system machine (Ingenia, Philips Healthcare, Best, Netherlands) was used for volumetric imaging (1.0 mm), with the 3DT1 MPRAGE and 3DT2 FSE sequences. A LightSpeed VCT 64-slice CT scanner (General Electric Company, Boston, MA, USA) was used for thin-cut (0.65-mm) axial CT images.

### 3D Rendering

All 3D planning and modeling studies were performed with Mimics Innovation Suite 22.0 Software (Materialise, Leuven, Belgium). DICOM files (in all axial, coronal, and sagittal planes) were imported into Mimics. The masking process was undertaken using Hounsfield unit (HU) values on 2D radiological images, and segmentation of various anatomical structures was performed according to defined anatomical borders visualized on MRI scans. Bone was segmented based on CT scans, whereas MRI was used for all other structures (i.e., soft tissue). The 2 imaging modalities were merged and aligned with the Align Global Registration module.

**Figure 2:**
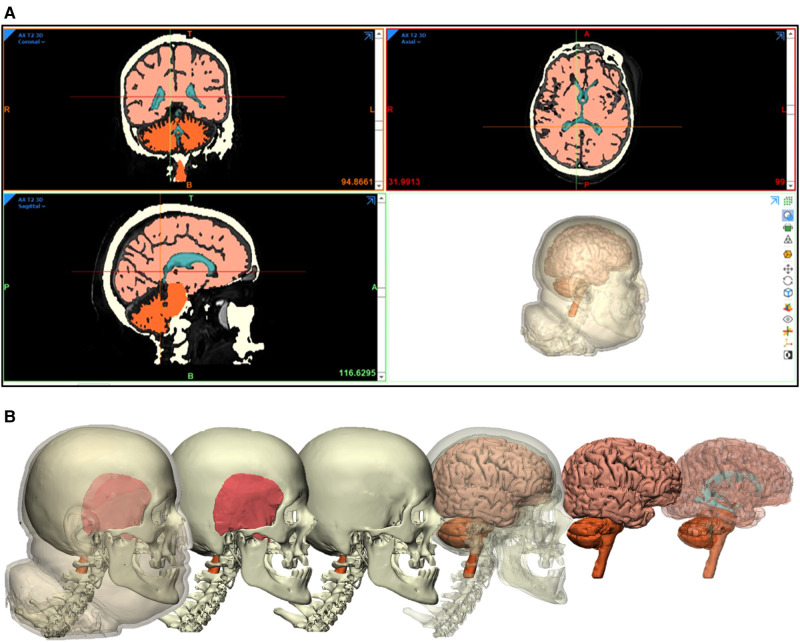
Segmentation and rendering process. (**A**) Neuroimaging data used for 3D rendering of segmented anatomical structures. (**B**) 3D rendering of segmented anatomical structures. *Used with permission from Barrow Neurological Institute, Phoenix, Arizona*.

Three-dimensional surface-rendered models of different anatomical structures were created. The design module feature (3-matic 14.0, Materialise, Leuven, Belgium) was used for fine-tuning and model details. Segmented structures included the skin, temporal muscle, bone, cerebrum, cerebellum, brainstem, and ventricles (Figure 2).

### Pterional Craniotomy Model

**Figure 3:**
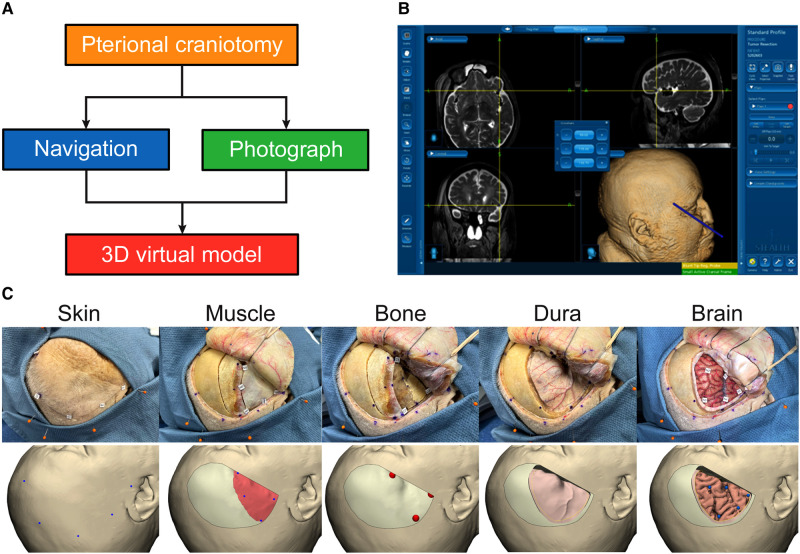
Creation of virtual pterional craniotomy model. (**A**) Steps to create 3D pterional craniotomy model. (**B**) Synchronous data acquisition through neuronavigation. (**C**) Stepwise pterional craniotomy with images in the real-world setting (top panels) and 3D virtual model (bottom panels). *Used with permission from Barrow Neurological Institute, Phoenix, Arizona*.

To create a 3D pterional craniotomy model, neuronavigation used during a stepwise surgical procedure was combined with photogrammetry (Figure 3A).

### Surgical Procedure

A cadaver head was placed and fixed in the Mayfield 3-pin head holder. Neuronavigation registration was performed using surface landmarks (Figure 3B). A pterional craniotomy was performed in a stepwise manner. Surgery was divided into 5 steps: (1) skin incision and dissection, (2) muscle incision and dissection, (3) burr holes and craniotomy, (4) durotomy, and (5) the intradural phase (exposure of brain) (Figure 3C).

### Neuronavigation

Medtronic StealthStation S7 (Medtronic PLC, Minneapolis, MN, USA) was used for neuronavigation. Volumetric MRI scans were imported. Registration was performed using surface landmarks. Cartesian coordinates (x, y, z) were acquired and recorded for all registration points and assigned points in 3D space using the navigation probe. Each step of the surgical procedure included the acquisition of 3 to 8 different points corresponding to anatomical structures in the surgical field. For each point, screenshots of the navigation system were obtained verifying and illustrating the corresponding coordinates on axial, coronal, and sagittal planes as well as the 3D reconstructed image on the neuronavigation system (Figure 3B).

### Photography

All stages of surgery were photographed with a professional camera (Canon EOS 5DS R, Canon Inc., Ota City, Tokyo, Japan) and a smartphone (iPhone 12, Apple Inc., Cupertino, CA, USA). Each acquired point in the surgical field was tagged with a 5 × 5-mm paper marker indicating an alphabetic-numeric code (e.g., S1 for the first point of skin incision) and photographed for further cross-validation.

### Importing Neuronavigation Data to the 3D-Rendered Virtual Model

After the surgery was performed and the Cartesian coordinates for each point were obtained and verified with navigation, all points were imported into a 3D virtual model environment. The original landmarks for neuronavigation registration were used to register the point cloud of the 3D virtual model space. Integrating neuronavigation coordinates and photography data allowed surgical steps to be simulated on the 3D model. All pertinent surgical steps and layers of a pterional craniotomy were included, such as skin incision, muscle incision, burr holes and craniotomy, dura incision, and brain exposure (Figure 3C). Points relevant to each step were added to the 3D virtual environment.

### Machine Learning–Assisted 3D Reconstruction of 2D Photographs

Traditional photogrammetry tools use multiple photographs of an object to create 3D representations, whereas a novel AI-based depth-estimation tool developed to produce accurate 3D reconstructions of single 2D photographs provides a new form of photogrammetry. Intel ISL MiDaS v2.1 (Intel Labs, Santa Clara, CA, USA) is a neural network–based platform for robust depth estimation from 2D images that was used to create depth maps. Depth maps are images where every pixel is assigned an intensity value on a specific color-range scale according to its location on the z-axis (i.e., depth axis). Open3D library (an open-source library (11)) was used to create a point cloud from depth maps. MeshLab software (Visual Computing Lab, Pisa, Italy) was used for quality improvement and simplification of 3D modeling and surface reconstruction. The Sketchfab (Epic Games, Cary, NC, USA) website platform was used to visualize and share 3D reconstructed photographic images.

### Quantitative Validation Studies

**Figure 4:**
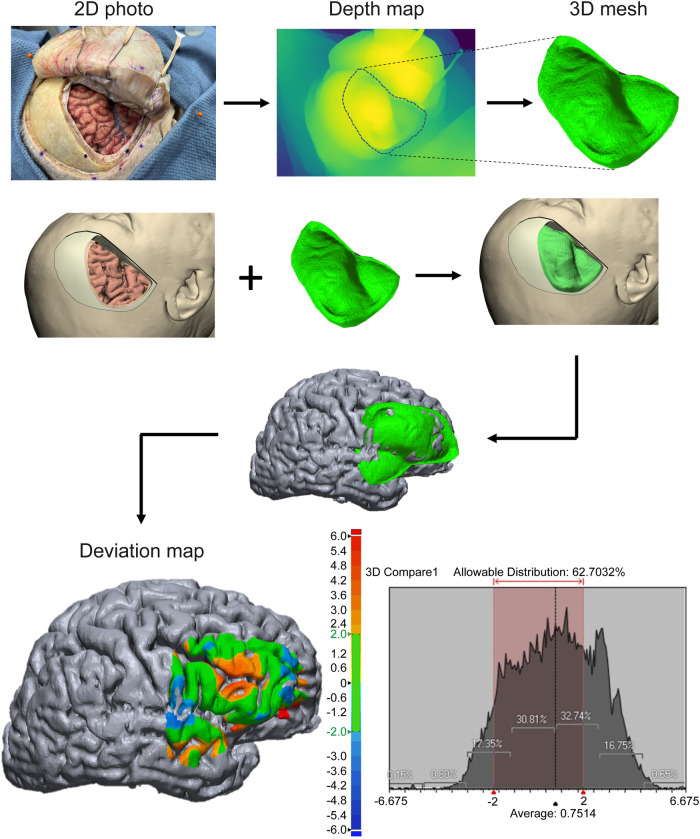
Validation of monocular-depth estimation technique using the 3D virtual model. A single operative photograph was used to create a depth map and corresponding 3D mesh. This mesh was then imported into the 3D virtual model space as an object. Two models (3D mesh of the photographic depth map and 3D virtual model) were aligned, and the deviation map between 2 surfaces was created. The deviation map shows that almost two-thirds of the entire surface area matches almost perfectly between the 2 models (within 2-mm limits). The epicenter and edges show the highest degrees of distortion. Colored scale bar indicates the degree of distortion in millimeters. *Used with permission from Barrow Neurological Institute, Phoenix, Arizona*.

In theory, neuronavigation and imaging-based 3D modeling should demonstrate inherently excellent spatial accuracy because of their reliance on high-resolution volumetric MRI and CT imaging. However, the validity of 3D reconstructed photographs has not been reported previously in the neuroanatomical literature. Therefore, we used the 3D space of the image-based reconstructed model as the ground truth and validation for other modalities (e.g., neuronavigation coordinates of the surgical field and depth maps of 3D reconstructed images) (Figure 4). In other words, the 3D reconstructed surface rendered from standard medical imaging served as the true reference values.

### Cross-Validation Studies

Pairwise comparisons of the two modalities were used for cross validation. Geomagic software (3D Systems, Rock Hill, SC, USA) was used to quantitatively measure how well two surfaces (neuroimaging-based 3D model vs. depth map of 3D rendered photograph) matched. The software also produced a color-scale deviation map (Figure 4). The use of neuronavigation also served as a valuable conduit to accurately rebuild the “invisible” anatomical details on the 3D virtual model (e.g., superficial sylvian vein, cortical arteries, etc.) by converting qualitative information of photography into quantitative data.

## Results

### Feasibility of the Methodological Pipeline

A variety of alternative photogrammetry and 3D-rendering methods were tested. Figure 1 demonstrates our proposed pipeline, which was found to be feasible and effective after a few iterations on different cadaver heads during the development phase.

### Generation of Neuroimaging-Based 3D Virtual Models

We created displays of successful reconstruction and segmentation of 3D virtual models, using volumetric imaging data (Figure 2). Highly accurate and realistic features of skin, bone, dura, and brain surface models were created. Sulci and gyri anatomy of the brain mirror those seen in photographic views.

**Figure 5:**
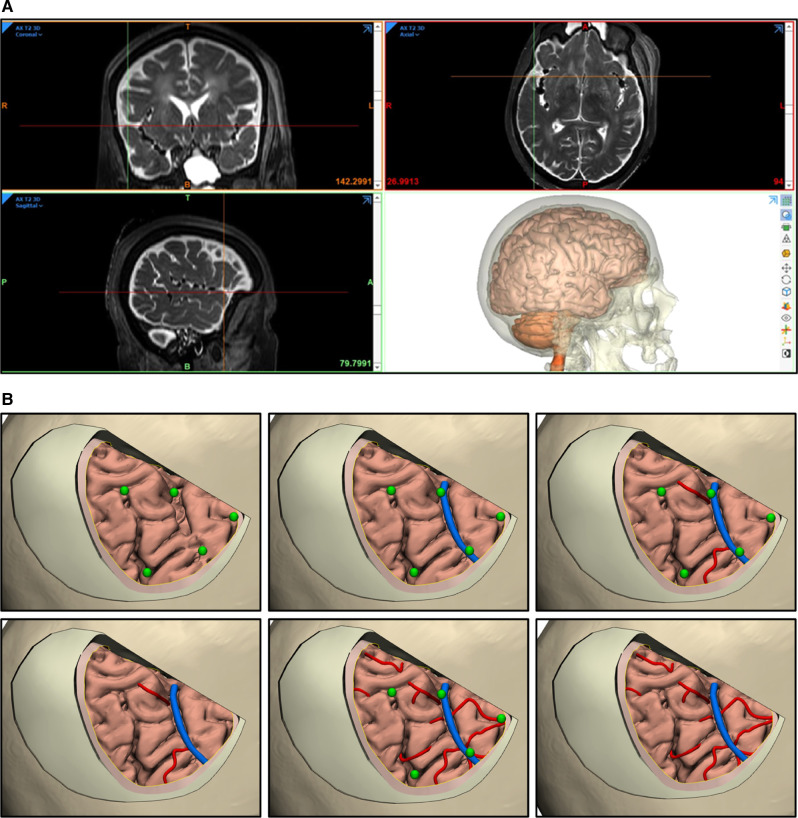
Adding vasculature to 3D virtual models. (**A**) Vascular structures are visible on MRI (coronal, top left; axial, top right; sagittal, bottom left). However, the delineation of vascular structures in 3D models (bottom right) is not practical or accurate unless a contrast medium is used before the vessels are injected. (**B**) An alternative method is to artificially add or draw arteries (*red lines*) or veins (*blue lines*) using certain anatomical landmarks acquired via neuronavigation and photography (*green dots*). *Used with permission from Barrow Neurological Institute, Phoenix, Arizona*.

Unlike in vivo human brains, cadaver neuroimaging lacks a clear visualization of vascular structures; therefore, this 3D virtual model does not show vasculature. However, the casts of injected major arteries and veins can still be traced and rendered manually with the software, or a contrast medium can be used for injection if desired. Alternatively, microvasculature or other fine anatomical details can be added manually by using photographic information (Figure 5).

### Cross-Validation of Neuronavigation with a Neuroimaging-Based 3D Model

**Figure 6:**
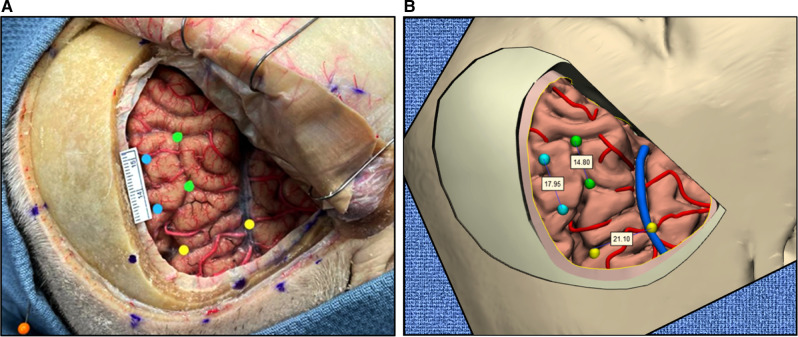
Measurements in the real-world setting using a photograph (**A**) correspond to the 3D virtual model (**B**). Ruler and values shown are millimeters. *Used with permission from Barrow Neurological Institute, Phoenix, Arizona*.

After we constructed an imaging-based 3D model, we assessed the accuracy and validity of the model using the neuronavigation tool in the real-world setting. Co-registration of the 3D space from the neuronavigation system with the 3D virtual model allowed us to quantitatively measure the distortions arising from the surgical procedures on a cadaver (i.e., brain shifting). We measured the differences between the “true” model (image-based 3D reconstruction) and the measured (navigation) coordinates of prespecified points at each step of the surgical procedure. Overall, mean distortion was calculated as 3.3 ± 1.5 mm (range, 0–7 mm) for all 29 points acquired during the surgical procedure. Measurements in the 3D virtual model perfectly matched (defined as deviation <2 mm) that of real measurements on cadaveric specimens (Figure 6).

### Validation of 3D Images Reconstructed from Single 2D Photograph

The validity of machine learning–based 3D reconstruction (a novel form of photogrammetry, using monocular-depth estimation) of a single 2D photograph was assessed. The first validation step involved analyzing the depth map (seen in Figure 4). The results showed that the depth map accurately represented relative depths of anatomical structures in the surgical field.

The second step was a quantitative analysis of the same depth map with reference to the 3D model’s cortical surface exposed through the pterional craniotomy. The mean (SD) distortion was 0.7 ± 1.9 mm (range, −6.7 to 6.7 mm). Overall, within the craniotomy window, 63% of surface maps were perfectly matched (deviation <2 mm). Notably, the central area (around the inferior frontal sulcus) showed the largest distortion, whereas the match at the peripheral cortical surface was nearly perfect (<2 mm distortions). However, distortions increased again toward the edges.

**Figure 7:**
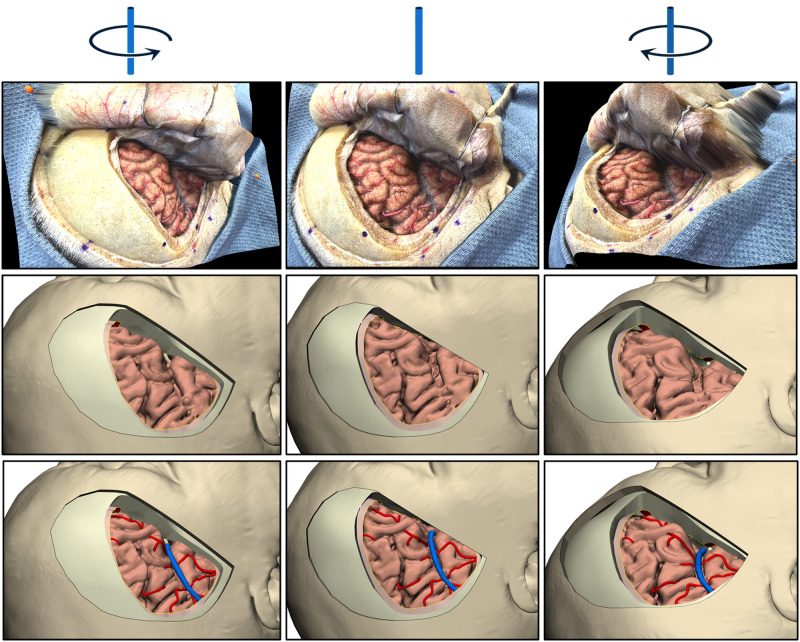
Three rotations (columns) of real-world and two 3D models. Cadaveric photographs are shown in the top row. Both the 3D images obtained from a single 2D photograph via monocular-depth estimation technique (middle row) and the 3D virtual model generated from neuroimaging data (bottom row) can be rotated and matched in 3D space. *Used with permission from Barrow Neurological Institute, Phoenix, Arizona*.

The third step was qualitative validation. Real-time inspection of the 3D images created by texture mapping yielded accurate 3D perception in a wide range (up to 45° in all directions from the perpendicular axis of neutral image). Furthermore, qualitative validation was demonstrated objectively by comparing the same angle visualization of both the 3D photograph and the 3D virtual model (Figure 7).

### Feasibility of Multimodality Integration

Our findings support the feasibility and applicability of the virtual models to be used as simulation tools for education, research, and surgical planning. The capability of supplementing the 3D model with anatomical details invisible to neuroimaging is demonstrated in Figures 5–7.

## Discussion

In this study, we introduced and validated a novel methodological pipeline that integrates various imaging and modeling technologies to create an immersive cadaveric simulation. We combined cadaveric dissections, virtual reality, surgical simulation, and postdissection analysis and quantification. Our findings suggest the following. First, neuroimaging and 3D modeling technologies can successfully digitalize precious cadaveric materials with a breadth of volumetric information and segmentation options. Second, various forms of photogrammetry technology can supplement these 3D models with more realistic surface information, such as fine anatomical details, color, and texture. Third, neuronavigation can be used as a medium to connect, communicate, calibrate, and eventually combine these two 3D technologies. Lastly, all these technologies could potentially pave the way for more integrated and immersive neurosurgical simulations. To the best of our knowledge, ours is the first neuroanatomical study to show the feasibility of combining the spatial accuracy provided by standard medical imaging (i.e., CT and MRI) with overlaid realistic textural features to a 3D reconstructed model.

### Cadaveric Dissection as a Training Tool

Historically, cadavers have been considered the best material to use for studying human anatomy from both training and research perspectives. Also, cadaveric dissection is the gold standard for training neurosurgeons because it allows surgical techniques to be demonstrated, provides a unique experience with a wide range of sensory inputs, and creates necessary surgical skills (12, 13). These skills include depth perception, 2D and 3D vision orientation, sensitive movements in limited environments (14), bimanual coordination, and hand-eye coordination (15, 16). In fields such as chess, music, sports, and mathematics, studies on human performance have shown that attaining an expert performance level requires about 10,000 hours of focused practice (17, 18). To obtain 10,000 hours of practice in a certain field, a person would have to dedicate 5 hours/day, 6 days/week, 48 weeks/year for 6.9 years (18). This equates to the time needed to graduate from a neurosurgical residency in the United States (7 years). A significant percentage of hours can be efficiently spent in a cadaveric dissection laboratory. However, access to such laboratories is limited due to a global shortage of cadavers, increasing costs of cadaver materials, and fewer dedicated anatomy laboratories, as well as the recent coronavirus pandemic. All these factors have contributed to decreased availability and use of cadavers worldwide (19–23). As a result, these recent limitations have paralleled the rapid adoption of new technologies to replace cadavers in anatomical training and international collaborations to maximize the use of the existing cadaver supply (19, 20, 24–26).

### Virtual 3D Models and Simulators for Training

Three-dimensional virtual models are not unique to the field of neurosurgery or medicine. Use of these models began in the 1950s and 1960s as computer-aided design systems for military simulators and the aerospace and automobile industry (27–29). Later, mathematical algorithms were created to define virtual 3D solid models (27, 30). Now, with the emergence of advanced neuroimaging modalities and volumetric reconstruction, the means of teaching neurosurgical anatomy has been markedly influenced, opening the possibility for learning anatomy and simulating surgical interventions through virtual reality (13, 31, 32).

Neurosurgical education and training have been gradually moving toward virtual reality (5, 33). The COVID-19 pandemic further accelerated this trend. In fact, distant (i.e., remote) learning has become the mainstay of education during the pandemic (34). As a result, we believe now is the most opportune time to supplement neurosurgical training worldwide with 3D virtual reality.

The process of creating 3D virtual models in medicine requires the segmentation of CT or MRI, then volume rendering (35), which produces surface models with high spatial resolution. This technique is currently useful for identifying a pathologic location and surgical planning; however, the current 3D models lack color, texture, and fine anatomical details, such as fine blood vessels, cranial nerves, and arachnoid membranes, which cannot be reproduced (5). The next step in the evolution of 3D anatomical and virtual simulations is the development of models that are both spatially accurate and have fine anatomical detail and realistic textures.

### Photogrammetry as a Supplement to Anatomy Training

Neuroimaging-based 3D reconstruction models capture anatomical features with high spatial accuracy, but as noted above, they lack fine anatomical details and realistic textures. A possible solution to this limitation is to incorporate photogrammetry into building the models. Photogrammetry creates 3D representations using multiple 2D photographs of an object and has long been used in anatomy to bring 3D perception to 2D photographs or to create virtual 3D representations of anatomical specimens (1, 5, 10, 36–38). De Benedictis et al. (36) reported quantitative validation of photogrammetry for the study of white matter connectivity of the human brain. Although their study used a relatively sophisticated setup, our group recently showed the applicability of a freely available 360° photogrammetry tool for neuroanatomy studies (1). In addition, Roh et al. (5) also incorporated photogrammetry into a virtual 3D environment to create realistic texture details of cadaveric brain specimens, but their study lacked quantitative validity. Nonetheless, these studies clearly show the potential of this rapidly developing technology in enhancing neurosurgical and neuroanatomy training.

### Monocular-Depth Estimation as a Novel Form of Photogrammetry

Although photogrammetric technology has advanced tremendously in recent years, the various tools that are available continue to involve very complex hardware or software. However, the proposed monocular-depth estimation tool detailed in this study, which can also be regarded as a novel form of photogrammetry (Intel ISL MiDaS v2.1), appears to be a revolutionary game-changer. The technique provides spatially accurate features by AI-based and machine learning–guided 3D reconstruction of a single 2D image. When this tool is trained with a massive database of 3D films, it outperforms competing methods across diverse data sets (39). Indeed, we have demonstrated that this tool has a surprisingly high accuracy of estimating depth, even on a complex surface like the human brain. The potential for applying this novel photogrammetry tool in neuroanatomy training and research is enormous, and researchers will likely rapidly explore this technology in the near future.

### Integrating Various Technologies for Immersive Simulations

All the technologies mentioned in this study are valuable instruments on their own, but each has its own limitations. Nevertheless, each technology can potentially complement another’s weaknesses; therefore, when combined, they may offer an immersive, realistic simulation that is both quantitatively and qualitatively accurate. Thus, we believe the proposed methodological pipeline can be used not only for cadaveric dissections but also for real surgical scenarios. Images taken from surgical microscopes can be exported and implemented in our proposed pipeline. Some microscopes even support robotic and tracked controlled movements that can acquire stepwise stereoscopic imaging to postprocess into a 3D environment showing real tissue color, texture, and surgical approach anatomy (40). In addition, one can supplement the fine anatomical details that are usually missed when reconstructing standard MRI or CT images. This refinement is achieved using neuronavigation as a registration tool during cadaveric dissections and pinpointing the exact location of a structure that is “invisible” on neuroimaging.

Overall, we believe our proposed integrative approach can maximize the utility of cadavers and offers endless virtual dissection possibilities. In other words, a cadaver intended for dissection can be digitalized, so that it can remain an educational instrument forever rather than simply being destroyed when it can no longer be used. Physical models provide the advantage of real anatomical substances, but they are subject to decay and manipulation and require preservation and constant maintenance. In contrast, virtual models can be shared electronically, yield joint diagnostics and collective expertise, and eliminate geographical limitations (27). High-quality neurosurgical training and education can be continued in the comfort of a training neurosurgeon’s home or office rather than a cadaveric dissection laboratory.

### Supplementing 3D Virtual Models with Cadaveric Dissection and Live Surgery

Given the proposed pipeline, in what ways can 3D virtual models supplement neurosurgical training through cadaveric dissection and live surgery in real patients? In theory, the 3D methods can apply to both cadaveric heads and live heads, but one is inherently more pragmatic. The construction of an accurate and realistic 3D virtual model requires time, physical (i.e., surgical) dissection, and multiple measurements of different anatomical structures using neuronavigation. Unless an anatomical structure is related to the underlying pathology of a live patient, it will not be exposed in surgery, and under no circumstance will unnecessary dissection be performed on a live patient to expose distant structures. In addition, live patients will have blood and cerebrospinal fluid (CSF) in the surgical field, brain shift due to CSF removal, and brain pulsations that can interfere with the quality and precision of intraoperative imaging and registration. Fixed, injected cadaveric heads do not have limitations created by the real surgical environment. Working with cadaveric heads creates no immediate time limit. Cadavers can be dissected to show all desired anatomical structures. Also, using a cadaver creates no interference in measurements or registration caused by blood, CSF, brain shift, or pulsations as in live surgery.

Cadaveric tissue also has inherent limitations. Digitalizing a cadaver head before the irreversible physical dissection process allows for its repeated use via virtual dissections. This digital copy also provides an opportunity to continually improve the virtual model by acquiring cadaveric photos, even in standard 2D format with smartphones, professional cameras, or microscopes. While the actual cadaveric specimen is damaged from repeated use over time, it will give rise to a more complete and enriched digital cadaveric model dataset. Having such a dataset of cadaveric images and models will also enable novel anatomical measurements, simulation of different surgical approaches, and virtual dissections.

Although the purpose of surgical dissection in a live patient is to treat a certain pathology and not create a realistic 3D model, we believe that our proposed pipeline can be effectively used under certain circumstances in intraoperative situations without interfering with treatment. For example, serial surgical exposure images (both macroscopic and microscopic) can be co-registered with neuroimaging data using either neuronavigation registration points or more reliable anatomical landmarks (e.g., superficial veins). Then, machine learning algorithms can be used to train AI with the overwhelming intraoperative photographic data so that the algorithm can accurately predict the texture and surface details of MRI-based 3D-rendered virtual models.

However, it should be noted that surgical exposure will always be limited by a craniotomy performed at one specific time in a live patient, whereas a cadaveric specimen can be freely explored through many craniotomies and approaches at different time points. Furthermore, when using a cadaveric specimen, more aggressive retraction and dissection can be performed to reach distant anatomical targets that are deemed not safe in a real patient or are being tested for feasibility. Virtual models can support learning by allowing numerous rehearsals, with skills then confirmed by actual dissections in a laboratory. Eventually, with optimization of our pipeline and future advances in 3D virtual technology, we believe that accurate and realistic 3D virtual models can be effectively applied to both cadaveric and real-life specimens. Our technology is developed and exists to augment neurosurgical training; we have no doubt that considerable practice dissecting the cranium and brain is mandatory to achieve technical excellence for the progressing neurosurgeon.

### Study Limitations

This study describes and validates a novel methodological pipeline for neurosurgeons and neuroanatomists worldwide. It includes both modifications in already known technologies (3D modeling, navigation, and photogrammetry) and the introduction of new technology (AI-based monocular-depth estimation) into the field. However, the model has certain limitations. This model development pipeline is supported by our preliminary, proof-of-concept study and should be validated with a larger number of specimens and by other groups. The process requires some expensive materials and tools, such as cadavers, neuroimaging, neuronavigation, and modeling software. However, we believe this methodology can be implemented in laboratories that already have these capabilities and resources, and those laboratories could then share their resources and outputs with others across the world who are in need of these valuable and innovative educational materials. Although merging 2 technologies to complement each other provides an exciting opportunity, this process may not be as easy as thought due to the complexity of underlying technical substrates and operational principles of each technology. This potential training tool will require the collaborative effort of surgeons, anatomists, imaging scientists, software developers, computer vision and image recognition specialists, and even graphic designers. The image processing procedures described in this study still require human effort and expertise, which restricts their efficiency but allows for personalization, revision, and modification. AI applications will likely generate more automatic or semiautomatic processes in the near future.

## Conclusions

Our report represents the first time multiple 3D rendering technologies have been integrated and validated for the fields of neuroanatomy and neurosurgery. We were able to successfully merge the 3D reconstructions from standard medical imaging (CT and MRI) and photogrammetry (including a novel technique not yet applied to any form of anatomy) with high accuracy. As a result, we produced a 3D virtual model that is both quantitatively and qualitatively accurate. We believe our methodological pipeline can supplement both neurosurgical training and education globally, especially in a setting where cadaveric dissection or operating room access is limited. With 3D virtual technology, we envision a future where neurosurgeons can learn relevant anatomy and practice surgical procedures in the comfort of their own homes and at the pace of their choosing.

## Data Availability

The original contributions presented in the study are included in the article/Supplementary Material; further inquiries can be directed to the corresponding author.
